# Acquiring submillimeter-accurate multi-task vision datasets for computer-assisted orthopedic surgery

**DOI:** 10.1007/s11548-025-03385-2

**Published:** 2025-05-14

**Authors:** Emma Most, Jonas Hein, Frédéric Giraud, Nicola A. Cavalcanti, Lukas Zingg, Baptiste Brument, Nino Louman, Fabio Carrillo, Philipp Fürnstahl, Lilian Calvet

**Affiliations:** 1 Research in Orthopedic Computer Science, University Hospital Balgrist, University of Zurich, Zurich, Switzerland; 2 Computer Vision and Geometry, ETH Zurich, Zurich, Switzerland; 3 Institut de Recherche en Informatique de Toulouse, Toulouse, France

**Keywords:** Open orthopedic surgery dataset, 3D reconstruction, Feature matching, Surgical navigation, Surgery digitization

## Abstract

****Purpose**:**

Advances in computer vision, particularly in optical image-based 3D reconstruction and feature matching, enable applications like marker-less surgical navigation and digitization of surgery. However, their development is hindered by a lack of suitable datasets with 3D ground truth. This work explores an approach to generating realistic and accurate ex vivo datasets tailored for 3D reconstruction and feature matching in open orthopedic surgery.

****Methods**:**

A set of posed images and an accurately registered ground truth surface mesh of the scene are required to develop vision-based 3D reconstruction and matching methods suitable for surgery. We propose a framework consisting of three core steps and compare different methods for each step: 3D scanning, calibration of viewpoints for a set of high-resolution RGB images, and an optical method for scene registration.

****Results**:**

We evaluate each step of this framework on an ex vivo scoliosis surgery using a pig spine, conducted under real operating room conditions. A mean 3D Euclidean error of 0.35 mm is achieved with respect to the 3D ground truth.

****Conclusion**:**

The proposed method results in submillimeter-accurate 3D ground truths and surgical images with a spatial resolution of 0.1 mm. This opens the door to acquiring future surgical datasets for high-precision applications.

**Supplementary Information:**

The online version contains supplementary material available at 10.1007/s11548-025-03385-2.

## Introduction

Computer vision tasks are widely used in orthopedic surgery for various applications, including surgical navigation [1], robotic-assisted surgery [2], and the creation of surgical digital twins [3]. Computer vision enables real-time alignment of intraoperative optical images with preoperative 3D models of the anatomy [4], facilitating precise navigation of anatomical structures, including hidden substructures, for both surgeons and robotic systems. 3D reconstruction of the anatomy and feature matching are examples of typical tasks required by computer-assisted orthopedic surgery (CAOS) systems. Accurate solutions to these tasks have the potential to eliminate the need for markers, which are associated with a complex workflow [5]. Surgical digital twins also benefit from advances in 3D reconstruction, allowing high-fidelity replica of real-world surgery. These 3D reconstructions can, for example, be used for education, where they can provide medical students and surgeons with interactive and virtual environments, and to train surgical robots in highly realistic simulations [6].

The development of these computer vision methods requires large, realistic surgical datasets with accurate 3D ground truths. While extensive datasets exist for man-made environments [7], the medical field lags behind due to ethical and logistical challenges. Existing surgical datasets focus on minimally invasive surgery (MIS), and available open surgery datasets lack the realism and accuracy needed for precision applications [8]. This work addresses these gaps by working toward a method to acquire realistic ex vivo datasets with highly accurate 3D ground truth of the anatomy, represented as a surface mesh, and optical images with precise corresponding camera poses.

The contributions of this work are a comparative analysis of methods for acquiring an accurate surface mesh of the visible anatomy, a comparison of different calibration techniques to obtain camera poses, and a marker-based method for registering the surface mesh with the posed images, together with a method to assess the accuracy of each of these steps. Each proposed step yields very high accuracy, and therefore, our work promises significant potential for capturing realistic ex vivo surgical datasets. We also provide a pilot dataset, validated using a pig torso to simulate scoliosis surgery, and use this dataset to evaluate state-of-the-art (SOTA) surface reconstruction methods in sparse or dense viewpoint scenarios. The code and dataset can be found under https://github.com/emmamost26/CAOS3D_v0.

## Related work

**Anatomy surface reconstruction** CT and MRI, while excellent for preoperative imaging, are challenging and impractical for intraoperative 3D reconstruction. CT exposes patients to ionizing radiation, making repeated use undesirable, and MRI requires a magnetically controlled environment, limiting compatibility with standard surgical tools. Moreover, both modalities lack real-time imaging capabilities and are not typically available in operating rooms, adding logistical and cost challenges. Ultrasound (US), though real time, is operator-dependent and requires direct contact with the anatomy. In contrast, optical cameras are the preferred solution for 3D reconstruction of the visible anatomy. They provide real-time, radiation-free imaging and capture anatomical details without requiring physical contact.

Optical image-based methods like structure from motion (SfM) and simultaneous localization and mapping (SLAM) have been adapted for surgical applications, with some focusing on endoscopic images to map and track anatomy in real time [9]. Deep learning-based approaches, such as neural radiance fields (NeRF) and transformer-based stereoscopic depth perception, have enhanced surgical scene reconstruction [10]. Structured light techniques have also been explored but are less suitable for real-time applications due to narrow depth of field and slow acquisition speed [11]. Despite progress, most 3D reconstruction methods focus on MIS, highlighting a gap in open surgery methods that this work aims to address.

**Anatomy tracking** Marker-less tissue tracking, primarily explored in endoscopic surgery, often registers a preoperative surface mesh with early intraoperative data. Notable works include [12], which tracked heart motion in MIS, and [13], which used stereo-cameras for real-time tissue tracking during partial nephrectomy. In open surgery, [4] developed a method for marker-less registration of preoperative lumbar spine models using RGB-D data from an overhead stereo camera. Despite its promise, the method’s accuracy is limited by the unrealistic cadaveric dataset used, which does not fully replicate real surgical conditions. Improving precision necessitates the collection of more realistic datasets as highlighted in [8].

**Fig. 1:**
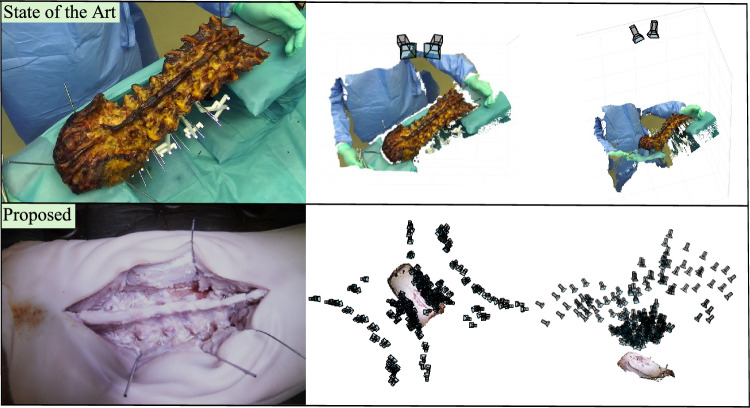
Comparison of our dataset acquisition method with SpineDepth [14]. SpineDepth offers limited viewpoint diversity (2 camera poses), cadaver images that are unrealistic for surgery, a mean target registration error of 1.5 mm, and a median deviation between ground truth and measured anatomy of 2.4 mm. Our method allows for unlimited viewpoints (216 in our experiments), and realistic images with a mean radial registration error of 0.35 mm

**Datasets with 3D ground truth** Large datasets containing posed images and 3D ground truths of indoor and outdoor man-made environments [15, 16] are a crucial prerequisite for data-driven 3D reconstruction [17, 18] and feature matching [19] methods, which have demonstrated clear superiority compared to traditional approaches [18, 20]. Recent trends in MIS have also pushed the publication of endoscopic datasets, some of which provide 3D ground truth and annotated poses [21] and are thus also suitable for surface reconstruction. However, datasets that feature only man-made scenes do not address the complexities of surgical data and MIS datasets are unsuitable for open surgery due to anatomical differences and lower image quality.

To the best of our knowledge, the only existing open surgery dataset is SpineDepth [14], which provides posed RGB-D images and the 3D scene geometry of dissected lumbar spines. However, the reported ground truth accuracy of 1.5 mm is insufficient to train pixel-accurate feature matching methods or high-quality surface reconstructions. Further limitations include the unrealistic exposure of the anatomy and a very limited number of viewpoints, as shown in Fig. 1. In contrast, our work proposes a methodology for the automated capture of high-quality images with submillimeter accuracy of both camera poses and the surface mesh of the anatomy. The method is designed specifically for surgical applications, supporting the development and evaluation of marker-less 3D reconstruction and tracking techniques.

## Methodology

In this section, we describe our proposed acquisition method to collect an accurate surface mesh of the scene registered to posed images with submillimeter accuracy. By *scene*, we refer to the specimen placed on an operating table, along with a set of 3D markers, consisting of spheres with known radii, fixated around it.

Our method comprises three steps, namely the scene surface reconstruction (Sect. Scene surface reconstruction), the capture of posed images (Sect. Capturing posed images), and the registration of the posed images with the surface of the scene (Sect. Scene registration). Separating the acquisition process into these three steps provides modularity and enables the comparison of state-of-the-art solutions for each step.

### Scene surface reconstruction

CT scanning is a well-established gold standard for acquiring 3D ground truth models of anatomical structures. Modern CT scanners achieve high spatial resolutions, making them ideal for capturing intricate anatomical details. For our CT baseline, we perform a CT scan on the animal specimen with a spatial resolution of 0.4 mm$^3$ (NAEOTOM Alpha, Siemens, Germany). The anatomy is segmented using Mimics (Materialise, Leuven, Belgium), followed by the extraction of a surface mesh.

However, CT scanning comes with several limitations: it is costly, not always easily accessible, and presents logistical challenges. Additionally, for the capture of an annotated dataset, transporting the anatomy between a wet lab or an operating room to an imaging center can introduce deformation, compromising the accuracy of the dataset. These limitations motivated us to compare CT to optical scanning, which eliminates the need to move the anatomy during the data capture. In this study, we utilize the *Space Spider* handheld 3D scanner (Artec 3D, Luxembourg), which offers a high point accuracy of up to 0.05 mm and a spatial resolution of 0.1 mm, making it a promising alternative to CT scanning. Note that the scene is scanned such that the positions and geometries of the markers are captured in the mesh. These are then used for scene registration, as described in Sect. Scene registration.

### Capturing posed images

Data capture can be performed manually or using a robotic arm. Manual capture requires minimal hardware and is most versatile, while mounting the camera on a robotic arm allows for automation. This second option is chosen for its scalability in surgical ex vivo data acquisition.

Camera poses can be obtained either using SfM [22, 23] or, if a robotic arm is used, using the robot’s forward kinematics. We evaluate these two approaches for camera pose estimation. The camera pose estimation based on the robot’s forward kinematics involves determining the transformation between the camera *C* and the robot’s end-effector *EE*, referred to as $T_{C}^{EE} \in \mathbb {R}^{4 \times 4}$ in the sequel. The camera pose can be expressed in the fixed coordinate frame of the robot’s base *B* as

1 $$\begin{aligned} T_{C}^{B} = T_{EE}^{B} \cdot T_{C}^{EE}, \end{aligned}$$

where $T_{EE}^{B}$ is the Euclidean transformation from the robot’s end-effector to base coordinate frame. The calibration of $T_{C}^{EE}$ is detailed in the Online Resource 1.

To enable a fair comparison between the SfM and robot-based camera pose estimation approaches, we evaluate both approaches on the same set of images captured with the camera attached to the robot arm.

### Scene registration

**Fig. 2:**
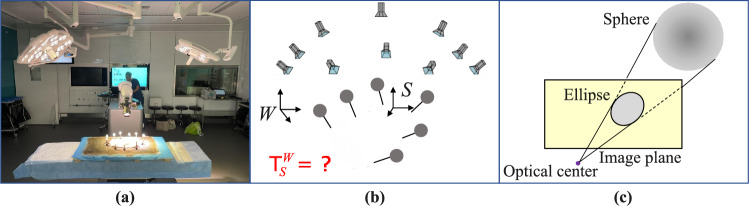
**a** Scene registration on a pig spine surgery in real operating conditions. **b** Given a set of *N* posed images of the scene expressed in the world reference frame *W* and a surface mesh of the scene expressed in a local reference frame *S*, the scene registration consists in recovering the relative pose $\textsf{T}_S^W$. We rigidly attach *M* spherical markers to the scene and fit spheres to the corresponding regions in the surface mesh to estimate their positions in *S*. **c** The spherical markers project into the image as ellipses, which are automatically detected and used to recover $\textsf{T}_S^W$

The final step involves registering the posed images with the surface mesh using 3D printed spherical optical markers rigidly affixed around the anatomy (see Fig. 2). These spheres are precisely localized in the surface mesh by fitting virtual spheres of the same radius to the mesh vertices using the iterative closest point (ICP) algorithm, with manual initialization. The precisely known positions and geometry of the markers make them reliable for accurate registration, compensating for the inherent limitations of SfM, which typically produces poses on a non-metric scale. Note that these markers are used solely for scene registration, not for camera pose estimation.

Inspired by [24], we perform a target-based registration from images of spheres to have the surface mesh of the scanned scene and the posed images expressed in a common reference frame. Given a set of posed images of a scene expressed in world reference frame *W* and a surface mesh of the scene expressed in the local reference frame attached to the scene *S*, the goal is to determine the relative pose $\textsf{T}^{W}_{S}{\in \mathbb {R}^{4 \times 4}}$.

**Optimization Objective** A sphere can be represented as a quadric matrix $\textsf{Q} \in \mathbb {R}^{4 \times 4}$, which is symmetric and defined as a function of its radius and center $\textbf{c}_\textsf{W} = \textsf{T}^{W}_{S}\textbf{c}_\textsf{S}$. It projects into the image as an ellipse $\textsf{E}$ of equation:

2 $$\begin{aligned} \textsf{E}^{-1} = \textsf{P}\textsf{Q}^{-1} \textsf{P}^T, \end{aligned}$$

where $\textsf{P}\in \mathbb {R}^{3 \times 4}$ is the camera projection matrix. Let $\textbf{x}$ denote the augmented Cartesian coordinates of a point on the ellipse in the 2D image plane. Any such point satisfies:

3 $$\begin{aligned} \textbf{x}^T \textsf{E} \textbf{x} = 0 . \end{aligned}$$

We take this bilinear product as the cost for our minimization problem and solve it using the Levenberg–Marquardt optimization method. Summing this cost over all *N* images, *M* markers, and a chosen number of ${L}$ points on the ellipse, the minimization can be written as follows:

4 $$\begin{aligned} \mathop {\mathrm {arg\,min}}\limits _{{\textsf{T}^{W}_{S}}} \sum _{i=1}^{N}\sum _{j=1}^{M}\sum _{l=1}^{L}\Vert \textbf{x}_{ijl}^T \textsf{E}_{ij}\textbf{x}_{ijl} \Vert _2^2 \end{aligned}$$

where $\textsf{T}^{W}_{S}$ represents the rigid transformation from the local coordinate frame *S* of the surface mesh to the coordinate frame *W*, in which the posed images are expressed. When the input poses are computed using SfM, a scale factor is jointly estimated for the camera poses. The extraction of ${L}$ points on the ellipse outline is detailed in Online Resource 1.

**Initial Estimates** The initial estimate for $\textsf{T}^{W}_{S}$ is computed with a Perspective-n-Point (PnP) solver, using the centers of the ellipses and corresponding centers of the 3D spheres as 2D-3D correspondences in one image of the image collection for which all the markers are well visible. Each images sphere is matched to its corresponding 3D sphere by exhaustively solving the PnP problem for all combinations of four selected ellipse centers paired with the *M* 3D sphere centers.

**Fig. 3:**
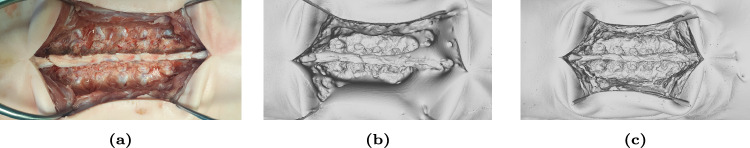
**a** RGB image of the open spine of a pig. **b** Reconstruction without coating. **c** Reconstruction with coating. The reconstruction with the spray clearly captures more detail, demonstrating the benefit of using the spray for improved surface reconstruction

## Experiments and results

### Acquisition protocol

We evaluated our proposed methodology in a simulated *ex vivo* scoliosis surgery using a pig spine. The specimen was rigidly fixed onto a wooden board with K-wires. An incision mimicking a scoliosis surgery was made by a clinician and held open using additional K-wires. 3D printed spherical markers (30 mm diameter) were affixed to a wooden board. Before collecting optical image data, the setup was transported to the imaging center for CT scanning. Afterward, it was returned to the operating room, placed on the operating table, and positioned alongside a robotic arm (LBR Med 14 surgical robot arm, KUKA AG, Germany) with a high-resolution camera (Alpha 7R V with a FE 24–70 mm F2.8 GM lens, Sony Group Corporation, Tokyo, Japan) mounted on its end-effector. The camera was focused on the scene’s center and its internal calibration was performed. Subsequently, $N=108$ images were captured from two robot positions on opposite sides of the operating table, including 30 viewpoints specifically selected to ensure optimal marker visibility for the scene registration. A video of the setup is provided in the Online Resource 2. Note that the dataset images do not necessarily contain the markers, ensuring that they accurately represent a realistic human surgery scene. Images containing visible markers can be cropped to exclude them. Due to the high initial resolution ($9504\times 6336$ px), cropped images retain a sufficiently high-resolution and optimal realism, as highlighted in Online Resource 1.

Following image acquisition, Aesub Blue scanning spray (AESUB GmbH, Germany) was applied to the anatomical surface to reduce reflectivity and enhance scanning quality, similar to the approach described in [25] (see Fig. 3). Finally, the entire scene, including the markers, was scanned with the optical scanner.

### Scene surface reconstruction

**Fig. 4:**
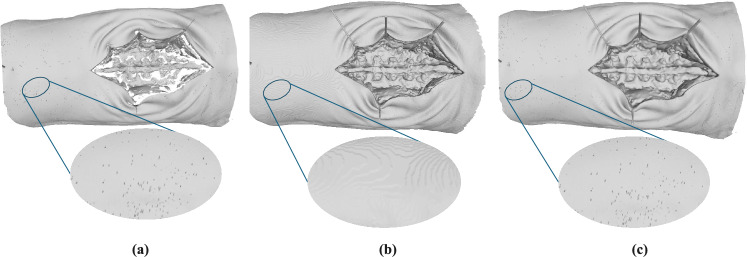
**a** The optical scanned mesh, obtained from the Artec3D Space Spider, captures very fine surface details, including the specimen’s hair. **b** The CT scanned mesh, with a resolution of 0.4 $\times$ 0.4 $\times$ 0.4 $\textrm{mm}^{3}$, is more effective at capturing concave areas, such as the interior of the wound, due to its ability to image internal structures. **c** A combined reconstruction using both the CT scan for concave regions and the optical scan for external surfaces, yielding the most detailed and accurate result

**Fig. 5:**
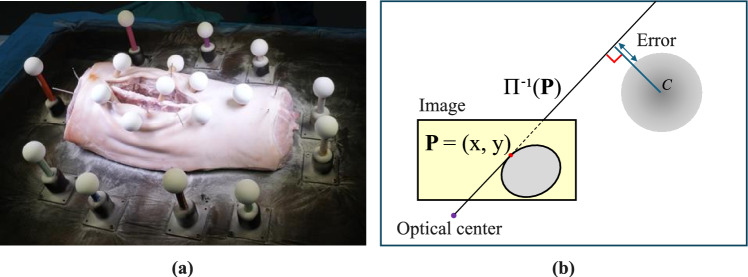
**a** Scene registration evaluation. Control markers were placed at the center of the scene. **b** We define the radial error to be the Euclidean distance between a ray back-projected from a point on the outline of the ellipse corresponding to an evaluation marker and the hull of the marker in 3D space

We provide a visual comparison of the reconstructions from the CT scan and the optical scan in Fig. 4. Low-frequency geometric features were accurately reconstructed with an average Chamfer distance of 0.7 mm between the optical scan and the CT-based model. High-frequency details were also well captured by the optical scan, as shown qualitatively in Fig. 4. While a CT scan of even higher spatial resolution might recover these details, the handheld optical scanner proved to be an excellent alternative. One drawback of the handheld scanner, however, is its partial reconstruction of concave regions, such as inside deep incisions.

We draw the following conclusions: (i) applying a coating is highly beneficial for improving scan quality; (ii) the optical scanner is a suitable and efficient alternative to CT for scanning anatomical geometries, except for deep concavities like wounds. For such regions, CT scanning remains the preferred option.

Finally, the surface meshes from CT scan and optical scanner can optionally be fused to obtain the optimal result in both concave and convex areas. To achieve this, one can either perform a 3D-to-3D registration of the spheres, as they are present in both scans and exhibit similar scanning quality, or run the proposed image-based scene registrations twice: one for the optical scan mesh and another for the CT scan mesh. In our experiment, we opted for the latter approach, aligning both meshes to a common coordinate frame, specifically that of the cameras. We then manually segmented the CT scan mesh to retain only the wound region and the optical scan mesh to retain only its outer region, while ensuring a few millimeters of overlap to avoid gaps in the final mesh. The two registered regions are then merged into a single mesh, resulting in the final ground truth mesh expressed in the cameras’ frame. This approach was used to create the pilot dataset, producing the anatomical mesh shown in Fig. 4c.

### Camera poses and scene registration

**Fig. 6:**
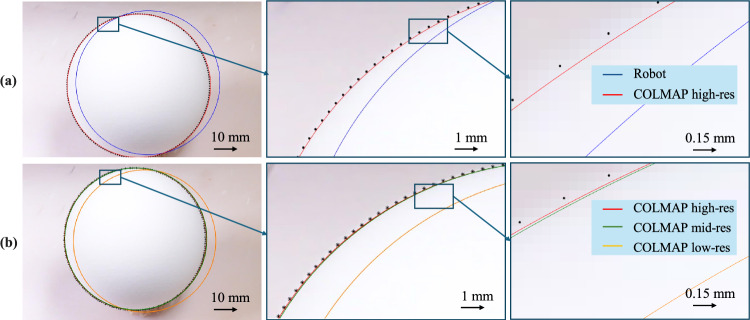
Qualitative comparison of the estimated camera poses from different modalities. **a** Using COLMAP with high-res images vs. using robot end-effector poses. **b** Using COLMAP with different image resolutions (respectively $9504\times 6336$ px, $4752\times 3168$ px and $1920\times 1080$ px). We obtained a mean reprojection error of 3.71 px using high-res camera poses from COLMAP against 8.99 px using robot poses. COLMAP with mid-res and low-res poses reported reprojection errors of respectively 3.91 px and 11.53 px

To evaluate the scene registration, we positioned spherical *control markers* on top of the specimen and inside the incision, distinct from the *M* = 10 *registration markers*, which were placed around the specimen (see Fig. 5). We captured *N* = 16 images from different viewpoints, scanned the scene with the optical scanner, and extracted the 3D marker locations, similarly to what was done in Sect. Scene registration. For each evaluation marker, we sampled points on the corresponding ellipse outline observed on each image and defined the radial error as the distance between a back-projected ray from a point on the ellipse and the corresponding 3D sphere, as depicted in Fig. 5. Reprojection error (in pixels) was calculated between the ellipse obtained by projecting the sphere into the image with the estimated scene registration solution and the detected ellipse. We compare the results across robot, COLMAP [22], and GLOMAP [23] poses in Table 1 and Fig. 6. Our analysis shows that using COLMAP poses with either high-resolution ($9504\times 6636$ px) or medium-resolution ($4752\times 3168$ px) images yields the most accurate results. While the accuracy with high-resolution images is slightly superior, the difference is minimal compared to the results obtained with medium-resolution images.

While using robot poses has the advantage of being independent of the scene appearance, it strongly depends on the accuracy of the poses delivered by the robot and requires that the robot base remains fixed during the entire data acquisition process, which makes it difficult to capture data from all angles.

**Table 1:** The scene registration method is evaluated in terms of radial errors and mean reprojection error (detailed in Sect. Camera poses and scene registration) for camera poses obtained from the robot, COLMAP, or GLOMAP

	Robot	COLMAP			GLOMAP
Image Resolution	–	Low	Medium	High	High
Mean radial error (mm)	0.89	1.10	0.37	**0**.**35**	0.44
Mean reprojection error (px)	8.99	11.53	3.91	**3**.**71**	4.67

The bold highlights the minimal error in each row, thus showing the best methods

For the SfM approaches, we compare the registration errors when using low ($1920\times 1080$ px), medium ($4752\times 3168$ px), and high-resolution ($9504\times 6336$ px) images

**Fig. 7:**
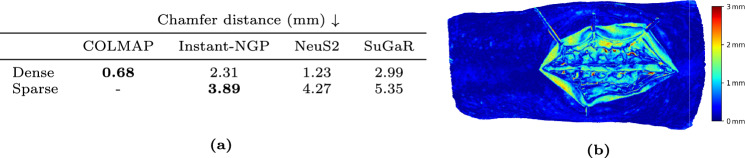
**a** Surface reconstruction errors (mm), measured by Chamfer distance (lower is better), are compared across two acquisition scenarios: (i) dense viewpoints (N=216) at a resolution of $3840\times 2160$ pixels, and (ii) sparse viewpoints ($N=8$) at $1920\times 1080$ pixels. COLMAP performs best in the dense viewpoint scenario but fails to reconstruct the scene with only N=8 viewpoints, in which Instant-NGP shows the best performance. **b** Heatmap showing the Chamfer distance between the model reconstructed with COLMAP and our ground truth

### Application to 3D reconstruction

We demonstrate one of the uses of our pilot dataset as a benchmark to compare different methods of 3D reconstruction from posed images. For this, we tested four methods that reconstruct the surface of a scene from RGB data, namely the traditional multi-view stereo method COLMAP [22], the NeRF-based methods Neus2 [26] and Instant-NGP [27], and the Gaussian splatting-based method SuGaR [28]. We evaluate them against our ground truth surface mesh. Note that the posed images used for the 3D reconstructions, as well as the 3D model serving as ground truth (Fig. 4c), are all expressed in the same coordinate frame. Consequently, the resulting 3D reconstruction can be directly compared to the proposed ground truth mesh. We evaluated the methods in a sparse scenario on mid-resolution images, using a subset (*N* = 8) of our captured images, simulating the use case of surgical navigation, where only few cameras are typically placed around the anatomy. We also evaluated them in a dense scenario (*N* =216) using high-res images, which would correspond to the use case of digitization using ex vivo specimens. The results are presented in Fig. 7. COLMAP achieves the best performance in the dense scenario, with a Chamfer distance to our ground truth of 0.68 mm, but it fails in the sparse scenario. Instant-NGP, however, holds in the sparse scenario, with a Chamfer distance of 3.89 mm, outperforming the other evaluated methods. Details on the computation of the Chamfer distance between the predicted meshes and the ground truth mesh are provided in Online Resource 1.

## Conclusion

We proposed a framework to acquire surgical datasets comprising an accurate surface mesh of the scene and posed images intended for the development and benchmarking of 3D reconstruction and feature matching methods. We evaluated various approaches for 3D scanning, recovering camera poses, and registering the scene along with the camera poses in an ex vivo scoliosis surgery experiment using a pig spine, conducted under real operating conditions. Based on these results, we proposed a combination that yields the most accurate results and is suitable for application on human specimens. Last, we demonstrated that a dataset captured with the proposed method is suitable as a benchmark for comparing different methods for 3D surface reconstruction.

## Supplementary Information

Below is the link to the electronic supplementary material.

Supplementary file 1 (mp4 1743 KB)

Supplementary file 2 (pdf 10185 KB)

Supplementary file 3 (mp4 26961 KB)

Supplementary file 4 (mp4 1743 KB)
