# Supplementary material for: Acquiring submillimeter-accurate multi-task vision datasets for computer-assisted orthopedic surgery
Source: Int J Comput Assist Radiol Surg. 2025 May 14;20(6):1293–300. doi: 10.1007/s11548-025-03385-2 (PMC12167284; doi:10.1007/s11548-025-03385-2)
Supplement: Supplementary file 2 — (pdf 10185 KB) [file 11548_2025_3385_MOESM2_ESM.pdf]

# Online Resource 1

## Acquiring Submillimeter-Accurate Multi-Task Vision Datasets for Computer-Assisted Orthopedic Surgery

Emma Most*, Jonas Hein, Frédéric Giraud, Nicola A. Cavalcanti, Lukas Zingg, Baptiste Brument, Nino Louman, Fabio Carrillo, Philipp Fürnstahl, Lilian Calvet

**Journal:** International Journal of Computer Assisted Radiology and Surgery (IJCARS)

**Affiliations of Corresponding Author:**
Computer Vision and Geometry Lab, ETH Zürich, Switzerland
Research in Orthopedic Computer Science, University Hospital Balgrist, University of Zürich, Switzerland

**Corresponding Author:** Emma Most
**Email:** `emmost@ethz.ch`

This supplementary material provides technical details and detailed analysis of the experiments that were omitted from the main paper due to space limitations.

# 1 Ellipse Detection

## 1.1 Spherical Marker Estimation

To get a first estimate of the spherical markers used for scene registration, we perform a bounding box detection, followed by segmentation and ellipse estimation from the obtained masks. The process is illustrated in Fig. 1.

*Sphere Detection* Sphere detection is performed using Grounding DINO [1], a model designed to detect arbitrary objects based on text inputs. We utilize the prompt "spheres" to find bounding boxes around the spheres in the image. If fewer spheres are detected than expected, we lower the confidence threshold to capture more uncertain detections, ensuring that the number of detected spheres meets a minimum count.

*Sphere Segmentation* Once bounding boxes are obtained, SAM2 [2] is used to segment the spheres within these regions. Starting from the center of each bounding box (assumed to be within the sphere), SAM2 produces a binary mask $m_c \in \{0,1\}^{w \times h}$ for each color channel $c \in \{R, G, B\}$, where $w \times h$ are the respective width and height of the bounding boxes. These masks are aggregated across channels by computing $m_{\text{agg}} = m_{\text{R}} \vee m_{\text{G}} \vee m_{\text{B}}$ and then postprocessing the resulting mask using morphological operations such as erosion and dilation. Only the connected component containing the center pixel $(\lfloor w/2 \rfloor, \lfloor h/2 \rfloor)$ is retained.

*Ellipse Fitting* Finally, ellipse fitting is performed to approximate the spheres, which appear as ellipses when projected to two dimensions. The ellipse parameters $(x_{\text{center}}, y_{\text{center}}, a, b, \theta)$, where $(x_{\text{center}}, y_{\text{center}})$ is the center, $a$ and $b$ are the semi-major and semi-minor axes, and $\theta$ is the rotation angle, are estimated by minimizing a loss function. This loss function $\mathcal{L}$ is defined as the sum of false positives (FP) and false negatives (FN), where an FP is defined as $\mathbf{e}_{ij} = 1 \wedge \mathbf{m}_{ij} = 0$, and an FN as $\mathbf{e}_{ij} = 0 \wedge \mathbf{m}_{ij} = 1$, with $\mathbf{e}$ being the binary mask of the fitted ellipse.

To improve convergence and avoid local optima, the regression is repeated with multiple starting conditions. The ellipse parameters that result in the lowest loss are chosen as the final estimate. If more ellipses are detected than expected, those with the highest losses are discarded, leaving the desired number of spheres. The final set of ellipse parameters provides a rough estimate and serves as a starting point for further pixel-level detection, described in Sec. 3.2 of the main manuscript.

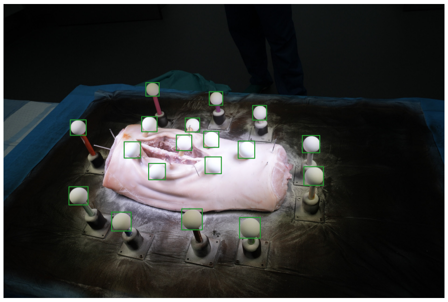

(a)

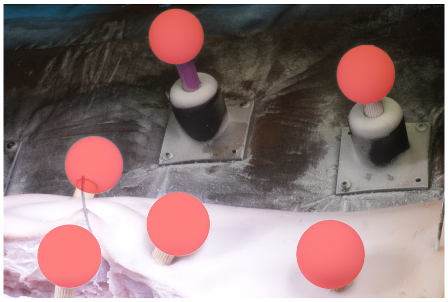

(b)

**Fig. 1** **a** Bounding boxes are extracted for all markers from a high-resolution image. **b** This serves as a basis for the segmentation and fitting of ellipses on the segmented masks

### 1.2 Extraction of Accurate Ellipse Edge Points

After obtaining an initial marker estimation, we detect Canny edges in the images and keep only the edge points contained in an envelope around the initial estimation. Then, we perform RANSAC to filter outliers and sample points equidistantly along the ellipse corresponding to the output of RANSAC. In our experiments, we sampled $K = 200$ points.

## 2 Camera and End-Effector Calibration

The camera intrinsics were recovered using the MATLAB Computer Vision Toolbox, assuming a standard pinhole camera model with two radial and two tangential distortion parameters. For calibration, we captured 90 high-resolution images of a professional checkerboard pattern[1] and obtained a mean reprojection error of 0.42 px for a resolution of $9504 \times 6336$ px. Throughout the calibration process and subsequent experiments, the focal length was fixed, and the aperture was set to its minimum (f/22) to maximize the depth of field and minimize blur. Shutter speed and ISO values were manually adjusted at the start to ensure proper exposure during data acquisition (ISO 100 and 1/15 s in our case).

The robot's end-effector to camera calibration was performed following an approach similar to [4].

[1] `https://calib.io/`

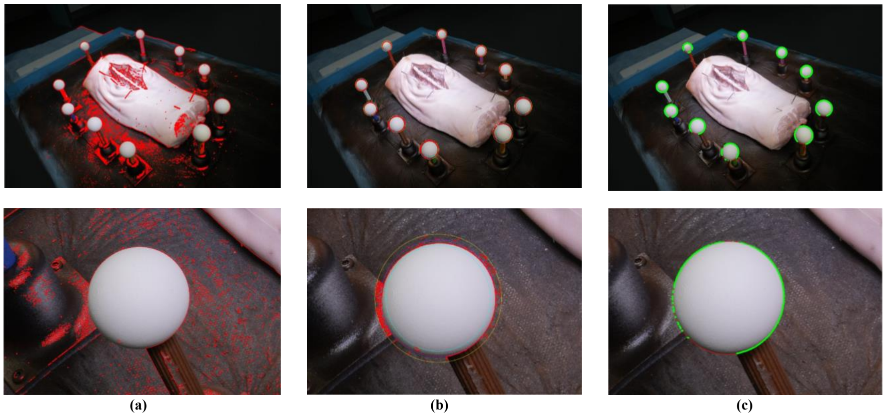


**Fig. 2** Extraction of accurate ellipse edge points, from left to right. **a** Edges detected by the Canny Edge Detector [3]. **b** Filtered edges contained in the region around the initial detection. **c** Inliers corresponding to the output of RANSAC

## 3 Marker design

In this section, we detail the design of the markers used for both scene registration and evaluation of the ground truth accuracy.

We employ 3D markers consisting of spheres mounted on rigid wooden cylinders, each being a few centimeters high and vertically fixed onto a wooden board where the specimen is placed. Both the spheres and the attachment bases of the wooden cylinders are 3D-printed. This design is chosen for its flexibility and precision, allowing easy adjustment of the markers' location and height to suit the size and shape of the specimen. These adjustments improve marker visibility within the camera's field of view and help reduce occlusions caused by the specimen. Figure 3 provides a close-up view of the markers affixed to the wooden board adjacent to the anatomy during data capture.

## 4 Evaluation

All quantitative evaluations are carried out using Chamfer distance between the reconstructed mesh $\mathcal{P}$ and the ground truth $\mathcal{G}$. For a reconstructed point $\hat{\mathbf{x}} \in \mathcal{P}$, its distance to the ground truth is defined as follows:

$$d_{\hat{\mathbf{x}} \to \mathcal{G}} = \min_{\mathbf{x} \in \mathcal{G}} \|\hat{\mathbf{x}} - \mathbf{x}\|, \tag{1}$$

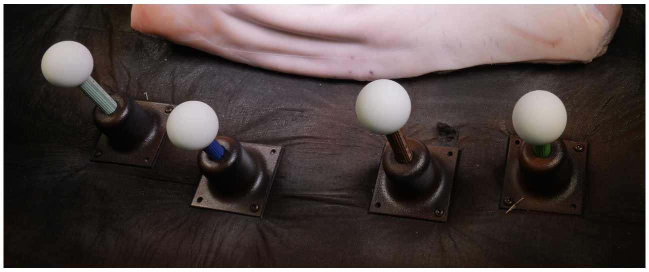

**Fig. 3** Up-close view of four markers used for scene registration.

and vice-versa for a ground truth point $\mathbf{x} \in \mathcal{G}$ and its distance to the reconstructed mesh. The distance measures are accumulated over the entire meshes to define the Chamfer distance

$$CD = \frac{1}{2}\left(\frac{1}{|\mathcal{P}|}\sum_{\hat{\mathbf{x}}\in\mathcal{P}} d_{\hat{\mathbf{x}}\to\mathcal{G}} + \frac{1}{|\mathcal{G}|}\sum_{\mathbf{x}\in\mathcal{G}} d_{\mathbf{x}\to\mathcal{P}}\right) \tag{2}$$

To minimize sampling error in the distance measurement (Eq. 1), the meshes were upsampled, maintaining a point spacing of 0.1 mm to adequately represent the surface geometry. Distances exceeding 20 mm were classified as outliers and excluded from the final score calculation similar to [5].

# 5 Dataset Samples

Figure 4 presents a sample image from our dataset with its associated depth map. For images containing markers, these can be removed by cropping while preserving high resolution. The resulting marker-free images remain accurately associated with the 3D ground truth, as demonstrated in Figure 5.

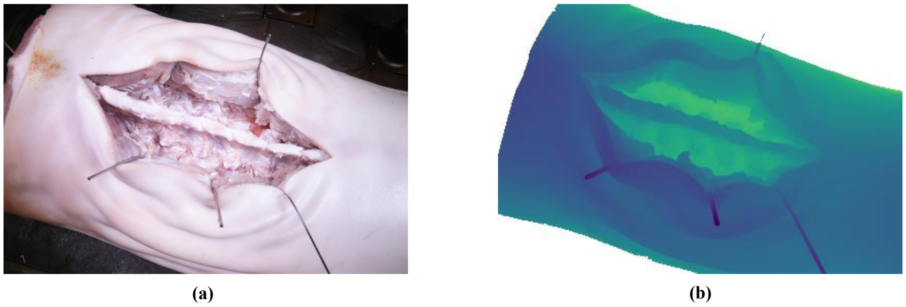

**Fig. 4 a** A sample of a typical image in our dataset, obtained with our method. It has a resolution of 9504 × 6636 pixels. **b** The associated depth map derived from our ground truth mesh

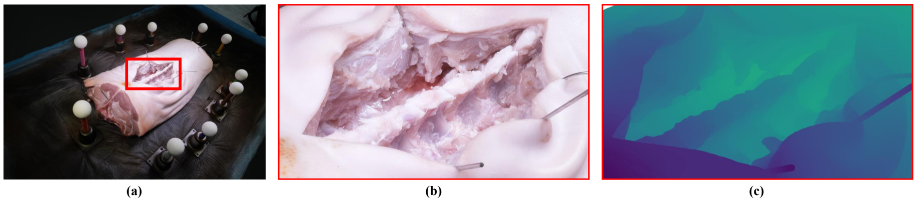

**Fig. 5** **a** Raw image of resolution $9504 \times 6336$ px. **b** Cropped image of resolution $1920 \times 1080$ px, which corresponds to a Full HD resolution. **c** Depth image derived from our ground truth mesh

# References


[1] Liu, S., Zeng, Z., Ren, T., Li, F., Zhang, H., Yang, J., Jiang, Q., Li, C., Yang, J., Su, H., *et al.*: Grounding dino: Marrying dino with grounded pre-training for open-set object detection. In: European Conference on Computer Vision, pp. 38–55 (2025). Springer

[2] Ravi, N., Gabeur, V., Hu, Y.-T., Hu, R., Ryali, C., Ma, T., Khedr, H., Rädle, R., Rolland, C., Gustafson, L., Mintun, E., Pan, J., Alwala, K.V., Carion, N., Wu, C.-Y., Girshick, R., Dollár, P., Feichtenhofer, C.: Sam 2: Segment anything in images and videos. arXiv preprint arXiv:2408.00714 (2024)

[3] Canny, J.: A computational approach to edge detection. IEEE Transactions on Pattern Analysis and Machine Intelligence **PAMI-8**(6), 679–698 (1986) `https://doi.org/10.1109/TPAMI.1986.4767851`

[4] Ozguner, O., Shkurti, T., Huang, S., Hao, R., Jackson, R.C., Newman, W.S., Cavusoglu, M.C.: Camera-robot calibration for the da vinci robotic surgery system. IEEE Transactions on Automation Science and Engineering **17**(4), 1762–1774 (2020) `https://doi.org/10.1109/TASE.2020.2986503`

[5] Aanæs, H., Jørgensen, R.R., Darkner, S., Henriksen, A.A., Jensen, R.T.: Large-scale data for multiple-view stereopsis. In: Proceedings of the IEEE Conference on Computer Vision and Pattern Recognition (CVPR), pp. 406–415 (2016)
